# HaloWeb: the haloarchaeal genomes database

**DOI:** 10.1186/1746-1448-6-12

**Published:** 2010-12-30

**Authors:** Satyajit L DasSarma, Melinda D Capes, Priya DasSarma, Shiladitya DasSarma

**Affiliations:** 1 Department of Microbiology & Immunology, University of Maryland School of Medicine, Baltimore, MD, USA; 2 Graduate Program in Life Sciences, University of Maryland School of Medicine, Baltimore, MD, USA

## Abstract

**Background:**

Complete genome sequencing together with post-genomic studies provide the opportunity for a comprehensive 'systems biology' understanding of model organisms. For maximum effectiveness, an integrated database containing genomic, transcriptomic, and proteomic data is necessary.

**Description:**

To improve data access and facilitate functional genomic studies on haloarchaea in our laboratory, a dedicated database and website, named HaloWeb, was developed. It incorporates all finished and publicly released haloarchaeal genomes, including gene, protein and RNA sequences and annotation data, as well as other features such as insertion element sequences. The HaloWeb database was designed for easy data access and mining, and includes tools for tasks such as genome map generation, sequence extraction, and sequence editing. Popular resources at other sites, *e.g.*, NCBI PubMed and BLAST, COG and KOG protein clusters, KEGG pathways, and GTOP structures were dynamically linked. The HaloWeb site is located at http://halo4.umbi.umd.edu, and at a mirror site, http://halo5.umbi.umd.edu, with all public genomic data and NCBI, KEGG, and GTOP links available for use by the academic community. The database is curated and updated on a regular basis.

**Conclusions:**

The HaloWeb site includes all completely sequenced haloarchaeal genomes from public databases. It is currently being used as a tool for comparative genomics, including analysis of gene and genome structure, organization, and function. The database and website are up-to-date resources for researchers worldwide.

## Background

Genomic data are essential resources for modern biology and are most useful when freely accessible to all. This is especially true when databases are curated and simple and efficient data mining tools are available. Major centralized repositories have been useful, and play a crucial role [1-5]. However, due to the complexity and diversity of genomic data, it is very difficult, if not impossible, to meet all scientific demands solely through these major repositories. Well-designed smaller, more specific (clade or family) databases and websites can be vital for analysis and research, especially for individual laboratories focusing on model organisms [6].

The first haloarchaeal genome sequenced was that of *Halobacterium *sp. NRC-1 [7,8]. Initially, the 191 kilobase pair plasmid pNRC100 was sequenced and made public in 1998 [7]. In 2000, with the sequencing of the remainder of the 2.57 megabase pair genome of NRC-1, the annotation of pNRC100 was extensively revised and updated [8]. To provide access to the most current data and facilitate functional genomic studies on *Halobacterium *sp. NRC-1, we created a custom database and website named HaloWeb. The prototype HaloWeb site was made available to the public in 2000 as a service to the community and has been available for the past ten years [6-21].

With the recent increase in the number of completed genomes, including ten additional haloarchaeal genomes [22-28], research efforts have shifted from the single- to the multiple-genome level. As a result, it became necessary to update the HaloWeb site to incorporate the newly sequenced genomes, including up-to-date annotation data. The updated HaloWeb site incorporates enhanced data access and mining tools for *Halobacterium *sp. NRC-1 and the other haloarchaeal genomes.

Among the onsite tools are those for genome map generation, gene and intergenic sequence extraction, and sequence editing, which have been developed and implemented on the website. In addition, other popular web tools and resources have been dynamically linked. The database and website also provide templates for additional on-going genome sequencing projects, and we expect to maintain and update resources for future data mining. Finally, the HaloWeb platform also provides an information management system to our laboratory for integration of public genomic data with additional proprietary transcriptomic and comparative genomic resources.

## Results and Discussion

**Figure 1:**
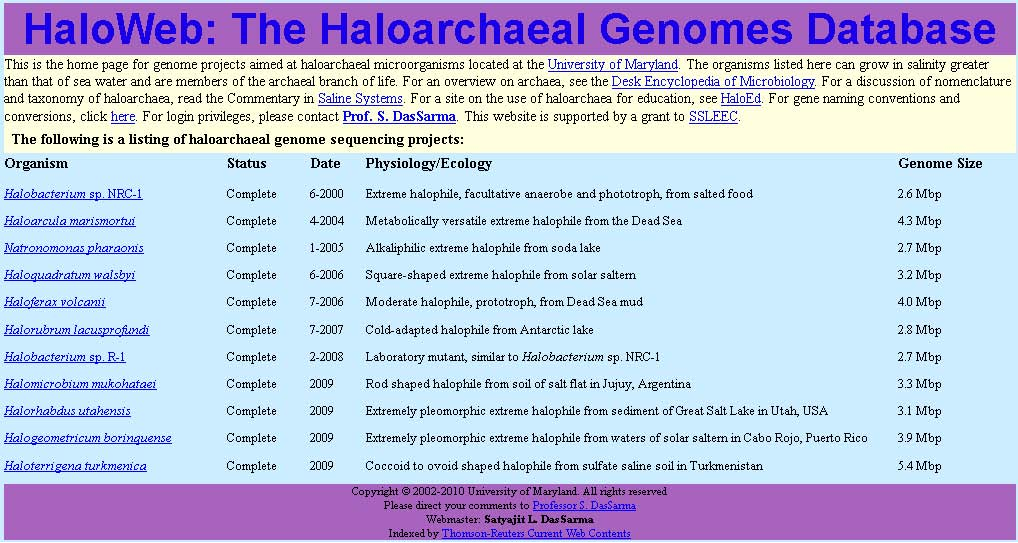
**HaloWeb Database Gateway Page**. This page provides information (sequencing status and date, physiology and ecology, and genome size) and links for the eleven sequenced haloarchaea.

The HaloWeb server has been established utilizing Free/Libre and Open Source Software (FLOSS) including the Linux, Apache, MySQL, and Perl (LAMP) stack [29]. The HaloWeb gateway page (Figure 1) contains links to the 11 haloarchaeal homepages, as well as other useful resources such as HaloEd, a database for education using halophilic microorganisms, and convention and conversion information. Most information is freely accessible in the public domain portion of the site.

### HaloWeb Genome Home

The genome homepage for each organism contains links to the organism's gene table, search page, and genome maps, along with the sequence editing tool, links to BLAST and genome sequence download pages in NCBI, as well as abstracts on the organism in PubMed.

### Gene Table

The gene table allows for genomic analysis of all 11 organisms by providing selection options using different criteria, such as replicon and gene type. Having a uniform interface for interaction also generates a consistent view, from which database transversal is facilitated. The gene table contains data for locus, orientation, replicon, annotation, and gene ID, for each gene.

### Search Tool

The search tool provides a comprehensive approach to data mining, allowing a search for genes based on ID number, name, annotation, or location in each genome. This is implemented using MySQL queries to the organism's database, optimized for quick retrieval by using the minimum columns necessary to complete the table, in a unified interface.

### Gene Page

**Figure 2:**
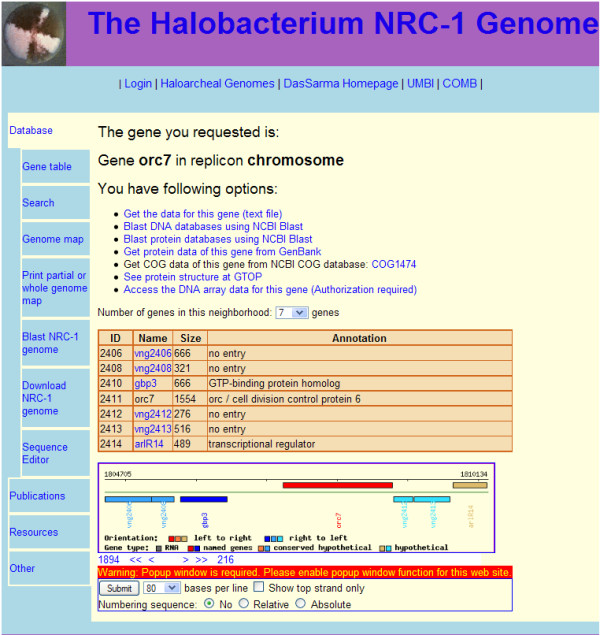
**HaloWeb Gene Page**. An example of a gene page is shown for *Halobacterium *sp. NRC-1 *orc7*. A variety of information (when available) is linked near the top of the page, followed by a table of the gene region, a corresponding genetic map, and sequence data form.

The HaloWeb gene pages (Figure 2) allow access, via links, to information resources for the gene using our custom query interface tools to the database. The tools permit BLASTing the gene against protein and nucleotide databases at NCBI, accessing protein data at GenBank [2], and accessing the associated COGs and KOGs from NCBI [30]. There are also links to the KEGG [3] and GTOP [4] databases. A table is also generated containing links to surrounding genes, the number of which may be selected by a dropdown menu. The table also contains each gene's ID, name, size, and annotation.

For an alternate way of navigating the database, a gene map with links to surrounding genes is available. The number of genes is regulated by the dropdown menu, and uses an image map to add informational popups and links to the otherwise static map. Controls below the map move the gene map window by changing the gene selected or by allowing leaps to either end of the current map. Below the map is a form containing controls for a popup with sequence data for the current gene region. Optionally, sequence data for an area around the gene, including intergenic sequences, can be retrieved.

### Maps

**Figure 3:**
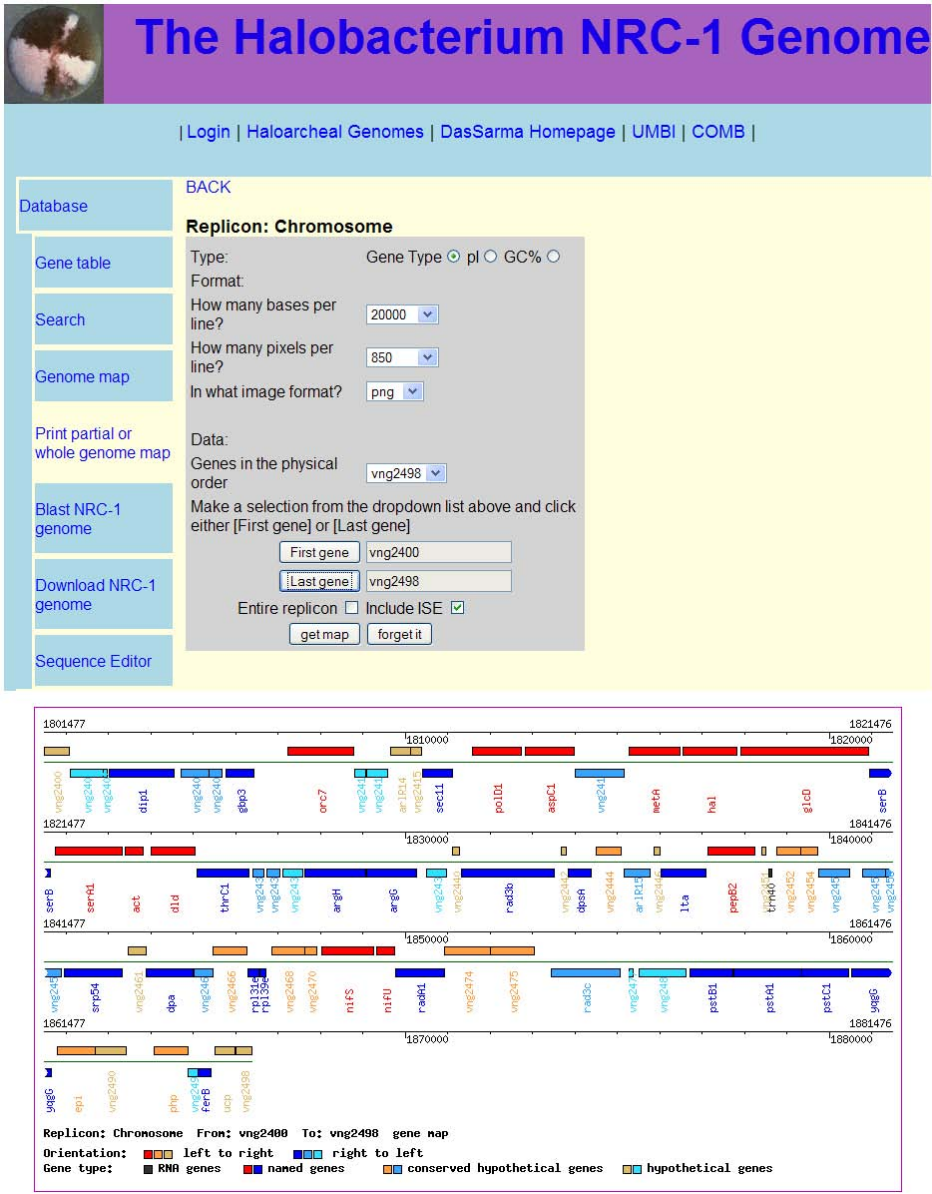
**HaloWeb Map Query Page**. An example of a map query is shown for the *Halobacterium *sp. NRC-1 chromosome. After input of the desired specifications, a map of the region is generated.

Map queries are also possible in HaloWeb (Figure 3). The first dialog is accessed by clicking on the "Genome Map" link. This dialog contains a replicon selection radio box and a button to continue to the next section. The second section is a form to set the format for the generation of the map, including dropdown menus for bases per line, pixels per line, and a list of genes. The list of genes is used for selecting the first and last genes, using buttons to fill in the read-only text boxes. There are also check boxes to use links or get the entire replicon. Finally, the map is generated by clicking the "Submit" button, which opens a new tab with the image.

## Conclusions

With the completion of the updated HaloWeb site, genome data from a major family of microorganisms, the haloarchaea, are readily accessible. This resource has served the academic research community for many years. In addition, HaloWeb also includes proprietary in-house generated data, including microarray and protein cluster data, and serves as a useful laboratory information management system [31].

## Methods

### Software Tools

Red Hat Enterprise and Fedora Linux, in both 32 bit and 64 bit versions, are used to run the servers. The Apache 2 web server is used to serve up web pages, and a MySQL Community server is used for the database backend. Most scripts are implemented using Perl, connecting to MySQL using the DataBase Independence (DBI) Perl module from Common Perl Archive Network (CPAN) as our database frontend, to allow the greatest flexibility in script writing and database program usage. The usage of the Perl language allows easy graphics generation by the GD library, such as the gene mapping utility, through the GD object-oriented module, and parameter passing is through the Common Gateway Interface (CGI) module. In some cases, JavaScript code is also utilized.

### Genome data

Genome data for the following organisms was obtained from NCBI: *Halobacterium *sp. NRC-1, *Haloarcula marismortui*, *Natronomonas pharaonis*, *Haloquadratum walsbyi*, *Haloferax volcanii*, *Halorubrum lacusprofundi*, *Halobacterium *sp. R-1, *Halomicrobium mukohataei*, *Halorhabdus utahensis*, *Halogeometricum borinquense*, and *Haloterrigena turkmenica*.

## Competing interests

The authors declare that they have no competing interests.

## Authors' contributions

The HaloWeb database structure was designed by SD, and software development and systems administration was provided by SLD. Database tables and website testing were provided by MDC and PD. The manuscript was written by SLD, MDC, PD, and SD. All authors read and approved the final manuscript.
